# Experimentally Created Magnetic Force in Microbiological Space and On-Earth Studies: Perspectives and Restrictions

**DOI:** 10.3390/cells12020338

**Published:** 2023-01-16

**Authors:** Svetlana A. Ermolaeva, Vladislav A. Parfenov, Pavel A. Karalkin, Yusef D. Khesuani, Pavel A. Domnin

**Affiliations:** 1 Gamaleya National Research Centre for Epidemiology and Microbiology, 123098 Moscow, Russia; 2 Institute of Metallurgy and Material Science, Russian Academy of Sciences, 119334 Moscow, Russia; 3 National Research Nuclear University MEPhI (Moscow Engineering Physics Institute), 115409 Moscow, Russia; 4 Institute of Cluster Oncology, Sechenov First Moscow State Medical University, 127473 Moscow, Russia; 5 3D Bioprinting Solutions Ltd., 115409 Moscow, Russia

**Keywords:** magnetic levitation, spaceflight, bacteria

## Abstract

Magnetic force and gravity are two fundamental forces affecting all living organisms, including bacteria. On Earth, experimentally created magnetic force can be used to counterbalance gravity and place living organisms in conditions of magnetic levitation. Under conditions of microgravity, magnetic force becomes the only force that moves bacteria, providing an acceleration towards areas of the lowest magnetic field and locking cells in this area. In this review, we consider basic principles and experimental systems used to create a magnetic force strong enough to balance gravity. Further, we describe how magnetic levitation is applied in on-Earth microbiological studies. Next, we consider bacterial behavior under combined conditions of microgravity and magnetic force onboard a spacecraft. At last, we discuss restrictions on applications of magnetic force in microbiological studies and the impact of these restrictions on biotechnological applications under space and on-Earth conditions.

## 1. Introduction

Microbiologic studies hold an important position in space exploration due to the significance of the role that bacteria play in human life. Considerable efforts have been made in microbiological studies related to human health (for a review see [1,2,3]). Another important trend in microbiology-related space studies deals with multiple practical applications from waste utilization to development of microbial cell factories under spaceflight conditions [4,5,6]. Significant efforts made in these areas have revealed noticeable changes in the metabolism and physiology of bacteria under spaceflight conditions. Spaceflight provides unique conditions of microgravity unavailable on Earth. Bacteria were shown to reply to microgravity by changes in growth rates, cell sizes, motility, biofilm formation, antibiotic resistance, and virulence [7,8,9,10,11,12,13,14]. In the last years, an increasing number of microbiological studies use “omics” technologies to analyze changes in gene expression, protein production, and metabolic pathways. Microgravity conditions change expression of stress response systems and factors, induce changes in genes associated with biofilm formation, affect surface structures including transporter proteins and pili, and noticeably affect spectra of secondary metabolites [15,16,17,18,19,20,21]. Besides the obvious importance of fundamental knowledge on changes in bacterial physiology and metabolism, applied applications of microgravity effects might be useful in future projects performed in space and on Earth and provide advantageous biotechnological platforms allowing for rational conversion of metabolic processes and improvement of bioprocessing efficiency. 

For more than 50 years, manned spacecraft and especially orbital stations Mir and the International Space Station (ISS) have provided onboard conditions for multiple microbiological experiments [1,22,23]. However, financial, safety and time restrictions on space studies have required development of on-Earth models to study specific microgravity features. For bacteria growing in liquid media, spaceflight conditions provide weightlessness, lack of sedimentation, and low shear conditions due to the absence of convectional flows within the liquid [1,24]. A number of on-Earth models were developed that mimic these hallmarks of microgravity. The devices based on rotation over one or two axes, designated depending on the construction as clinostats, random positioning machines (RPM) or rotating wall vessels (RWV) are widely used in simulated microgravity studies (for a review see [25,26,27]). These devices provide low fluid shear and lack of sedimentation. Cells placed in rotating devices undergo a combined force provided by the sum of the gravity and centripetal force vectors, which is characterized by a continuous change in the module and direction with the temporal average equal to zero. 

Another on-Earth simulated microgravity model is based on the effect of magnetic levitation, which is provided by balancing the gravity vector with magnetic force [28,29,30]. Magnetic levitation (MagLev) is widely used in applied studies to create conditions in which an object is suspended with no support other than magnetic fields to counteract gravitational effects. In microbiological research, MagLev allows for solving different tasks including cell separation, differentiation, or vice versa cell accumulation and integration depending on the system configuration [30,31,32]. Using MagLev systems in space studies is at its beginning, but application of the magnetic force under spaceflight conditions provides novel possibilities to modify bacterial growth conditions according to research and biotechnological necessity. In this review, we consider the physical nature of magnetic force, applications and restrictions on the magnetic levitation models and discuss how magnetic force affects bacteria under spaceflight conditions. 

## 2. Magnetic Force vs. Gravity

Magnetic force appears as a result of quantum effects of the non-homogenous magnetic field on atomic and molecular electrons. If the magnetic force affecting the diamagnetic object is upward directed and strong enough to counterbalance gravitational force conditions, (dia)magnetic levitation (MagLev) can be reached on Earth. Water and all living creatures are diamagnetic, which is why they can levitate in the magnetic field. Below, we give a short explanation of those aspects of magnetism that are important for understanding bacterial behavior in the presence of magnetic force. For a deeper view of the nature of magnetic force and magnetism, we address the reader to excellent reviews that contemplate these phenomena from physical and biological points of view [31,32,33,34,35].

When the external magnetic field is applied, magnetization of a certain material depends on its atomic properties, such as presence of unpaired electrons. Electron magnetic moments respond to the external magnetic field being attracted or pushed off. The magnitude of magnetization is quantitatively expressed via the magnetic susceptibility χ. Magnetic susceptibility χ of diamagnetic materials is negative (χ < 0) and have low values of approximately χ ≈ −10^−5^. Being placed in the magnetic field gradient, the diamagnetic object is pushed off to the area of the lowest magnetic field. In paramagnetic materials, the magnetic susceptibility is positive (χ > 0) and typically ranges from 10^−5^ to 10^−3^ [33,34]. When the external magnetic field is applied, paramagnetic objects are attracted to the area of a higher magnetic field similar to ferromagnetic but with incomparably less power. Thus, magnetic effects in diamagnetic materials is different from effects in paramagnetic materials.

The magnetic force gives an acceleration to any object equal to $a_{m}=\left(\frac{\chi }{\rho \mu_{{}^{\circ}}}\right)\frac{BdB}{dr}$, where χ is a magnetic susceptibility, ρ is density of the objects, μ_0_ is a vacuum permeability, **B** is a magnetic flux density, the value characterizing magnetic field at a given point of the working volume, and $\frac{dB_{}}{dr}\;$is its gradient at the same point. For diamagnetic objects, the magnetic force and acceleration $a_{m}$ is directed to areas with lower magnetic field.

The magnetic force acts throughout the object’s volume, so the force supporting the object within the magnetic field gradient is a volume integral of forces affecting internal structures according to their magnetic susceptibilities. Magnetic susceptibility of a cell is close to the susceptibility of water, which is an important part of the living cell. Guevorkian and Valles experimentally determined magnetic susceptibilities for unicellular eukaryotes *Paramecium* suspended in the water-based medium as χ_c_ = −0.911 × 10^−5^ and χ_m_ = −0.904 × 10^−5^ for cells and medium, respectively [36]. Magnetic susceptibilities of cell cytosolic and lipid fractions of the protist cell differ from magnetic susceptibility of water by 2% and 10%, respectively. Still, in some cases, the complicated composition of the living cell can affect the magnetic force distribution due to differences in magnetic properties of cellular structures. Mainly, a distinct response can be seen from specific intracellular structures with highly different magnetic susceptibilities, such as ferrimagnetic crystals of magnetotactic bacteria that have a magnetic susceptibility χ ≈ 10^6^ [37,38]. Besides such special structures, basic physical principles suggest that magnetic fields interact only weakly with biological matter itself [39,40]. Subtle effects associated with the presence of magnetic ions, such as iron Fe^3+^, magnesium isotope ^25^Mg^2+^ or zinc isotope ^67^Zn^2+^, can change rates of reactions catalyzed by enzymes, which include these metals in their active center and affect cell metabolism but do not provide forces able to move cells as wholes [41,42].

**Figure 1:**
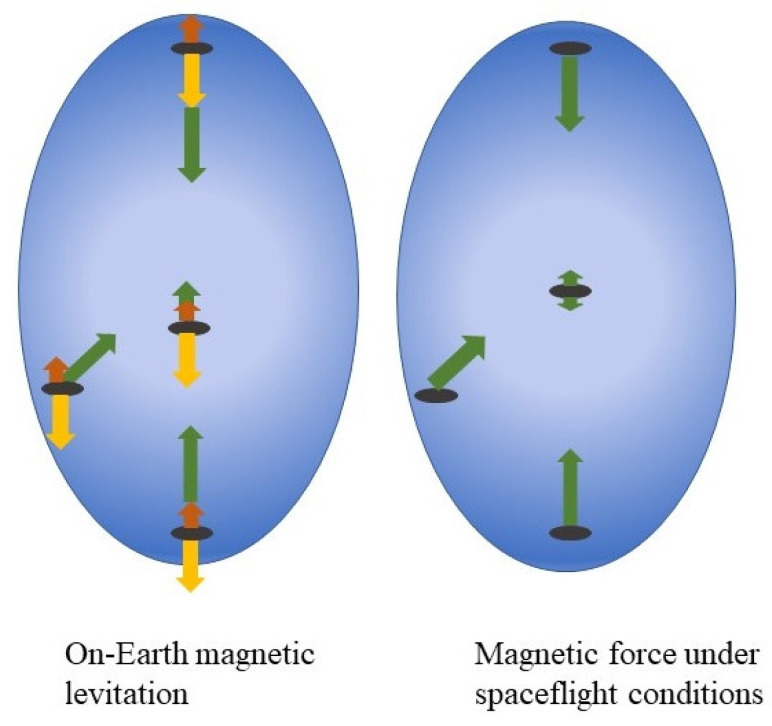
Forces that affect the bacterium in suspension within the non-homogenous magnetic field on Earth and under microgravity (spaceflight) conditions. On Earth, the bacterium experiences three forces, which are gravitational, Archimedes, and magnetic. Magnetic levitation is achieved at the point where the vector sum of the magnetic and Archimedes forces counterbalances gravity. Under spaceflight conditions, the magnetic force is the only force, it pushes the bacterium to the area of the lowest magnetic field. Green arrows—the magnetic force; yellow arrows—gravity; brown arrow—the Archimedes force. The gradient of the blue represents the magnetic field gradient: the magnetic flux density decreases as the distance from magnets increases and is the lowest in the center. (The figure modified [19]).

If we consider bacteria suspended in the liquid medium and placed within the non-homogenous CMF, the bacterial cell undergoes effects of three forces, which are gravitational, Archimedes and magnetic forces. To provide the MagL effect, the vector sum of the Archimedes and magnetic forces has to counterbalance the gravitational force (Figure 1). Among these three forces, the gravitational and magnetic forces operate throughout the volume, while the Archimedes force acts at the surface (for a detailed view, see [26]). In this way, magnetic levitation is different from the neutral buoyance that takes place for microorganisms floating in the ocean when the Archimedes force counterbalance the gravitational force and is closer to weightlessness. Dijkstra and co-authors experimentally evaluated values of the Archimedes and magnetic forces that affect *E. coli* suspended in a water-based medium placed in a superconducting magnet and demonstrated that these values are comparable [43]. For bacterial suspension in paramagnetic liquids (see below) the difference between the Archimedes and magnetic forces might be different than for water suspension although detailed data are unavailable yet. Therefore, the MagLev when applied to bacterial suspension provides partial weightlessness and can be applied to study the bacterial response on these conditions [44]. This fact together with numerous practical applications makes MagLev a very attractive method in microbiological studies.

Now consider forces affecting bacteria growing in the liquid culture onboard a spacecraft when the culture is subjected to the non-homogenous external CMF. All things onboard the spacecraft are essentially weightless. The weightlessness is due to the orbital centrifugal acceleration that balances the gravitational force putting all things in continuous “free fall”. The Archimedes force, which appears as a consequence of the difference between the cell’s weight and the weight of fluid displaced by the cell, is zero at these conditions. The magnetic force remains the only force that affects bacteria giving them an acceleration to move toward to areas with a minimal magnetic flux density (Figure 1). Therefore, the presence of a magnetic force changes conditions of bacterial growth at the spacecraft board where there are no other forces providing acceleration of the bacterial cell. From this point of view, an application of the magnetic force at the spacecraft board provides conditions of simulated gravity. If such a simulated gravity model is used some restrictions that result from the effects of the magnetic field itself, and its gradient should be taken into account. In the next sections, we consider systems used in microbiological studies to create a magnetic force, and their applications and restrictions.

## 3. Superconducting Magnets for Magnetic Levitation of Living Objects

Superconducting magnets used for magnetic levitation of living objects are organized as vertically oriented solenoids, with a bore ranging from 32 mm up to 195 mm, that provide strong spatially variated magnetic field reaching 33 T and more (for a review see [26,31]. Inside the bore, the magnetic field is strongest in the center of the solenoid and decreases upward and downward from the center. At the point of the strongest field, the gradient of the magnetic field is zero, therefore the magnetic force is zero, too. The magnetic force, which always directs to the area of the lower magnetic field, directs upward in the upper part of the solenoid and downward in the lower part. Levitation of diamagnetic material is observed above the center of the solenoid where the magnetic force counterbalances gravity. The values of the magnetic flux density at which levitation is observed ranges from 8 to 16 T depending on the solenoid particular configuration [29,43,45,46]. In contrast, the product of the magnetic field and its gradient $\frac{BdB}{dr}$ is theoretically calculated for a drop of water from the equation $a_{m}=g=\left(\frac{\chi }{\rho \mu_{{}^{\circ}}}\right)\frac{BdB}{dr}$, where the gravitational acceleration g = 9.8 m s^−2^, water density ρ = 1000 kg m^−3^, and water magnetic susceptibility χ = −0.9 × 10 ^−5^ [31]. This value is near 1370 T^2^ m^−1^ at the levitation point. The superconducting solenoid systems were successfully used to levitate frogs and their eggs, plants, protozoa and bacteria and to study their physiology and metabolism [29,36,43,45]. Still, such systems are not portative, especially when we think about space studies, and have restricted parameters of the of the levitation area (configuration of the magnetic force direction, square and the volume) [47]. 

A number of works investigated restrictions and artefacts generated by application of superconducting magnets in MagLev research that hide effects of simulated microgravity. The most important impact that might overshadow effects of weightlessness is the high magnetic field itself. Although superstrong magnetic fields (>10 T) were shown not to disturb exponential bacterial growth [48], such fields provide subtle effects on gene expression that affect bacterial growth at the stationary phase, cell morphology and production of secondary metabolites [48,49,50]. The magnetic field gradient can provide further effects on physiology and metabolism, although such effects were mainly described for mammalian cells and observed at high values of the gradient from 10^3^ to 10^9^ T m^−1^ [51]. Besides these direct effects associated with cell response on the magnetic field and its gradient, indirect effects changing bacterial growth were described. The magnetic field gradient forming in the superconducting magnets drives flows of oxygen that improves bacterial growth besides effects of weightlessness itself [43]. Potential gradients of nutritive substances and/or secondary metabolites expelled by bacteria have not yet been explored, although such gradients could affect the growth of bacteria. Supermagnet cooling systems affect temperature gradient that in turn raise convection flows that can also influence bacterial growth and is different from spaceflight microgravity conditions with the absence of convection flows [31]. Still, thorough studies showed that effects of the high magnetic fields and artificial microgravity can be separated, supporting suitability of the MagLev as a simulated microgravity model [50,52,53,54]. Taken together, these data suggest that the right controls play the most important role in experiments on magnetic levitation as a model to study microgravity effects on living objects.

## 4. MagLev Systems Based on Permanent Magnets

Strong permanent magnets made from rare earth element-containing alloys avoid some disadvantages of superconducting magnets. The particular benefits of permanent magnets include their relatively low cost, small sizes, independence on external infrastructure, and portability. Further, permanent magnets operate at lower magnetic fields and do not disturb uniform temperature distribution inside the working volume that prevents appearance of convection flows due to the temperature gradient. At last, diverse mutual arrangement of permanent magnets provides distinct spatial distribution of magnetic flux gradients to make it suitable for different tasks.

Neodymium-containing magnets, although the strongest among permanent magnets, do not allow for obtaining values of the magnetic flux densities higher than 2 T and cannot per se create a magnetic force sufficient for magnetic levitation [55]. The problem of low magnetic fields was successfully overcome for suspension of diamagnetic objects, such as bacteria by using paramagnetic or supermagnetic fluids [36,52,56,57,58,59]. The magnetic force affecting the diamagnetic object floated in the paramagnetic medium increases proportionally to the difference between magnetic susceptibilities of the cell and the medium [36,59]. Guevorkian and Valles experimentally demonstrated that the difference between magnetic susceptibilities of the unicellular eukaryotes *Paramecium* and standard cultivation medium is only 0.007 × 10^−5^, while supplementation of the medium with the 4 mM solution of gadolinium salt Gd-DTPA (gadolinium diethylene-triamine-pentaacetate) increases the difference 100-fold up to 0.764 × 10^−5^, which decreases the required value of the parameter $\frac{BdB}{dr}$ from 1370 T^2^ m^−1^ to 510 T^2^ m^−1^ [36]. Gadolinium is a rare-earth element widely applied in medical applications being included into complex chemical substances. Parfenov et al. demonstrated that supplementation of the liquid medium with another gadolinium-based substance, gadobutrol ([10-[2,3-dihydroxi-1-(hydroxymethyl)propil]-1,4,7,10-tetraazacyclododexan-1,4,7-triacetin(3-)-N1, N4, N7, N10,O1, O4, O7]gadolinium), taken in concentration of 10 mM provides levitation of human cells in the static magnetic field created with the NdFeB magnets with a remnant magnetization of 1.21 T [59,60]. Similar conditions allowed for magnetic levitation of bacterial cells [52]. The same system with the medium supplemented with 20 mM gadobutrol provides levitation of the macroscopic non-attached aggregates formed by bacteria and the bacterium-produced extracellular matrix [52]. MagLev in the 50 mM gadobutrol allowed for separation of viable and antibiotic killed *E. coli* according to their buoyancy [58]. 

Thus, medium supplementation with gadolinium derivatives provides magnetic forces sufficient for levitation of bacteria, bacterial aggregates, and mammalian cells using magnetic fields created by permanent magnets. However, when using these systems, potential toxic effects of the gadolinium salts should be considered as well as effects of the strong magnetic field and its gradient that are described above. Gadolinium derivatives are widely used as contrast agents in magnetic resonance imaging (MRI) techniques so their toxicity has been extensively studied [61,62,63]. An ability of the Gd^3+^ to compete with Ca^2+^ in active centers of Ca^2+^-binding enzymes underlies gadolinium toxicity because Ca^2+^ substitution with Gd^3+^ can interrupt critically important Ca^2+^-dependent pathways [61]. To decrease toxicity, gadolinium-based substances, which are used in MRI, enclose Gd^3+^ in the complex that is expected to remain chelated in the human body and be excreted intact. Fast and almost unchanged excretion was demonstrated for FDA approved contrast agents such as gadobutrol [62,63]. In bacteria, although Ca^2+^ ions do not play such a critical role as in mammals, Ca^2+^ dependent proteins take part in such cellular functions as motility and chemotaxis, virulence, and maintenance of membrane integrity [64,65,66]. Potential toxic effects of gadolinium-based substances should be taken into account in MagLev studies of bacterial physiology. 

Another limitation of the described approach is changes in the nature of the magnetic force action. The introduction of a paramagnetic fluid provides an effect similar to the effect of the Archimedes force, when a force is associated with displacement of the fluid volume. As a result, the force acts at the surface but not throughout the volume. Thus, although we obtain the neutral buoyancy, simulated microgravity is restricted. This effect limits using MagLev systems based on permanent magnets in studies of physiological changes in response to microgravity. On the other hand, permanent magnet based MagLev systems are effectively used in other applications.

## 5. Using the Magnetic Force for Cell Separation and Differentiation

**Figure 2:**
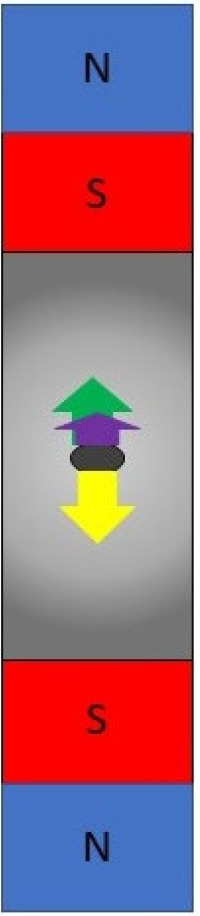
Scheme of the MagLev system used to separate suspended diamagnetic particles including bacteria. Arrows represent forces affecting the particle: green is a magnetic force; yellow is a gravitational force; violet is an Archimedes force. The gradient of the gray represents the magnetic field strength that decreases as the distance from the magnets. The particle levitates at the point where the vector sum of the magnetic and Archimedes forces counterbalances gravity. Particles are separated depending on their density.

The important benefit of permanent magnets is an ability to form different 3D patterns of the magnetic field gradient in accordance with the particular aim of the study by changing mutual arrangements of magnets. Depending on the system configuration, MagLev allows for solving different tasks, including cell separation, configuration or integration [30]. The basic MagLev scheme uses two straightforward magnets oriented to each other with the same poles [58,67] (Figure 2). This system has been improved by using parallel ring-shaped magnets or extended to use with 96-well plates [68,69,70]. Such systems are successfully used for cell separation studies. Distinct cell densities provide differences in Archimedes force that in turn affects requirements of the magnetic force balancing the cell, so cells with distinct densities levitate at different heights. Alternatively, levitation at discrete heights can be caused by distinct magnetic susceptibilities. Durmus and co-workers demonstrated that changes in bacterial membrane permeability caused by antibiotic treatment provide changes in levitation height that seems to be due to penetration of paramagnetic medium into the cytoplasm that in turn change both cell density and magnetic susceptibility [58]. Thus, the use of MagLev systems coupled with introduction of specific magnetic markers opens new perspectives in microbiological research.

To our knowledge, MagLev systems suggested for cell separation have not yet been used in space experiments. Under spaceflight conditions, the magnetic force is the only force pushing bacteria that would prevent cell balancing. Still, such systems could be efficiently applied in simulated gravity modeling in the course of space exploration along with centrifuges which are now used.

## 6. Using a Magnetic Force for Cell Integration and Aggregation

**Figure 3:**
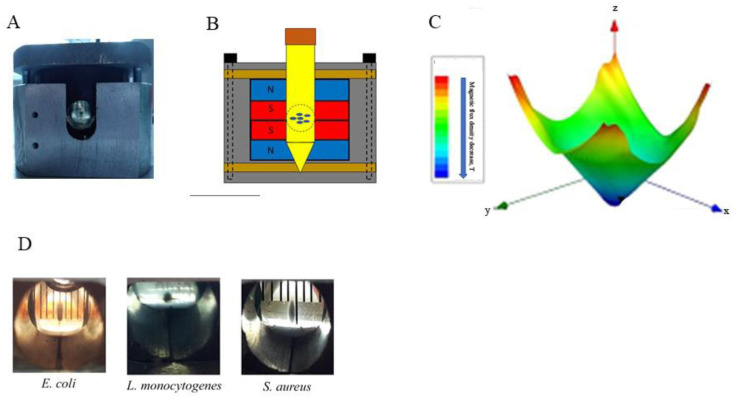
The MagLev system for bacterial aggregation. (**A**)—the magnetic bioassembler; (**B**)—the scheme of the bioassembler; (**C**)—the calculated magnetic flux formed within the bioaasembler [52]; (**D**)—non-attached aggregates formed by different bacterial species using the bioassembler.

While the above described systems are used for cell separation and differentiation, alternative mutual arrangement of magnets provides cell integration. Parfenov and co-authors reported a magnetic bioassembler that includes two round-shaped magnets located directly next to each other [59] (Figure 3). Such mutual arrangement of the magnets results in creation of a sharp gradient of the magnetic field decreasing by the center of the working volume where the magnetic field is the lowest. Bacterial and cell suspensions in the medium supplemented with gadobutrol, when placed into the bioassembler, gather near the center of the working volume [52]. Such an approach when applied to human cells provides scaffold-free, label-free and nozzle-free bioassembly of 3D tissues [59,71]. If applied to bacterial cells, the bioassembler stimulates a process of autoaggregation resulting in formation of non-attached bacterial aggregates [52].

Bacterial flocculation and autoaggregation within the liquid volume is a process typical for natural aquatic ecosystems, which is used industrially for waste-water treatments and other applications [72,73,74,75,76]. Non-attached aggregates formed by pathogenic bacteria are described in chronic infections [77,78,79,80]. Formed non-attached aggregates represent 3D multicellular structures that include bacteria and a self-produced matrix. Morphologically, such aggregates are similar to well-known biofilms. Biofilms are defined as surface-attached 3D multilayer structures, while non-attached aggregates float freely within the liquid bulk. Mechanisms of non-attached aggregate formation are relatively poorly studied because experimental studies are impeded by the lack of models preventing fast aggregate sedimentation. The described bioassambler has been successfully used to demonstrate that strains of well-forming biofilms might be deficient in non-attached aggregate formation, and vice-versa, non-attached aggregate forming strains do not form biofilms [52]. These data suggest that mechanisms underlying formation of biofilms and non-attached aggregates are different, at least partly. The MagLev approach provides a unique possibility to study molecular mechanisms underlying non-attached aggregate formation.

## 7. Magnetic Force Application in Space Studies

**Figure 4:**
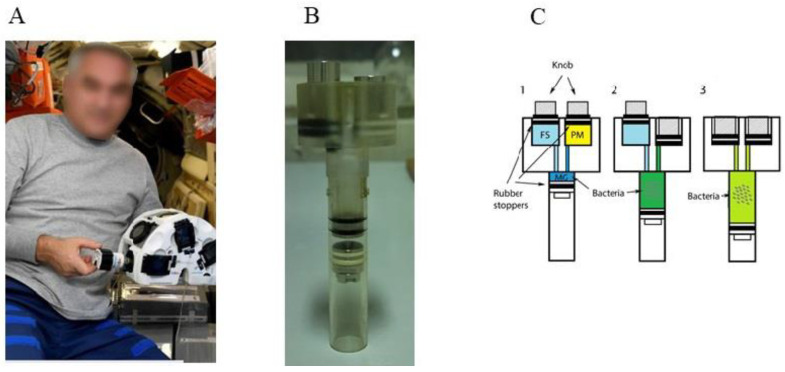
The magnetic bioassambler “Organ.Aut” used in space studies. (**A**)—the cosmonaut of Roscosmos, performing the experiment with “Organ.Aut” aboard the ISS; (**B**)—the working cuvette; (**C**)—the scheme of cuvette using: 1: before the start of the experiment bacteria were placed in the Melbiol hydrogel (MG), the nutritive paramagnetic medium (LB + 1 M Gadovist, PM) and fixation solution (FS) were in the isolated chambers; 2: then the cosmonaut pushed a button to mix MG and PM, and bacteria started to grow; 3: at the end of the experiment, the cosmonaut pushed a second button to fix bacteria; the total system hermetically sealed without air bubbles. Figure modified from [19].

The bioassambler “Organ.Aut” developed on the basis of described bioassambler was delivered to ISS at the end of 2019 [19,81]. “Organ.Aut” has six sockets each and includes two magnets arranged in the same way as in the ground bioassambler to allow for cell integration and aggregation, and with similar parameters (Figure 4). In the course of the space experiment, the bioassambler “Organ.Aut” was used to perform biofabrication of 3D structures under the combined impact of spaceflight conditions and the magnetic force [81]. In the bacterial part of the space experiment, the culture of the *Escherichia coli* probiotic strain M17 was placed in the bioassambler “Organ.Aut” [19]. To provide the magnetic force, the rich LB nutritive medium was supplemented with 20 mM gadobutrol. Bacteria in control cuvettes placed in “Organ.Aut” grew in LB without gadobutrol, so they experienced the magnetic field and its gradient but the magnetic force was negligible to provide bacterial accumulation.

The net effect of microgravity on bacteria may be distinct in its dependence on such parameters as a tested strain, medium composition or a growth stage (reviewed in [2,4,24,82]. Better availability of nutrients for non-sedimenting bacteria provide their better growth at exponential and early stationary growth. In contrast, absence of convective flows results in toxic by-product accumulation in the bacterial vicinity and exhaustion of nutrients and oxygen that is observed at later stages of growth [83]. Stimulated by the magnetic force bacterial accumulation in a small part of the working volume results in strengthening microgravity effects caused by the absence of convective flows. Particularly, bacterial response on nutrition and oxygen limitations during prolonged cultivation was more clearly manifested under combined conditions of microgravity and the magnetic force than conditions of microgravity only Proteomic analysis demonstrated that the response included the down-regulation of glycolysis and TCA enzymes and the up-regulation of methylglyoxal bypass that were more prominent under combined impact of microgravity and the magnetic force than microgravity only [19]. 

Autoaggregation is an established phenomenon observed in bacteria cultivated under real and simulated microgravity [8,9,17,84,85]. The bioassambler in space provided formation of visible macroscopic structures that included bacteria and non-cellular matrix and had morphology similar to morphology of non-attached aggregates obtained under MagLev conditions in the bioassambler on Earth [19,52]. Proteomic data revealed similar mechanisms that accompanied autoaggregation under conditions of real microgravity and MagLev [19]. Particularly, the surface protein Ag43 known to provide *E. coli* autoaggregation by direct protein-protein interactions was similarly up-regulated under all listed conditions [19,86]. Another feature similar for bacterial growth under MagLev and spaceflight conditions was upregulation of glyoxylate shunt enzymes and Vitamin B12 transporter BtuB [19]. Both the glyoxylate shunt and the transporter BtuB are active under anaerobic conditions and can be involved in utilization of metabolites released by destroyed bacteria [87,88,89,90,91,92]. The glyoxylate shunt is essential when acetyl-CoA is a direct product of metabolic pathways, for example, via degradation of acetate, fatty acids, and alkanes supplied by lysed cells [87,88]. The BtuB is the transporter of vitamin B12, which is an essential co-factor of ethanolamine deaminase, the enzyme involved in degradation of bacterial membrane phospholipids [92,93,94]. Utilization of metabolites supplied by lysed cells has to be effective in autoaggregates where dead and alive bacteria are in close proximity while it might be useless for a planktonic culture cultivated under standard gravity conditions when dead bacteria sank to the bottom. Thus, microbial response on low shear conditions and microgravity utilizes mechanisms developed to form non-attached aggregates on Earth. 

Establishment of a direct bacterial response on weightlessness (gravitaxis) besides indirect effects of low shear conditions accompanying absence of convective flows is a challenging task that has not yet been fully resolved [95]. From this point of view, the maximal interest would represent inverse effects of the magnetic force under magnetic levitation conditions and in space. Under MagLev conditions, the magnetic force counterbalances the gravitation force and the cell experiences partial weightlessness as it was discussed above. Under spaceflight conditions, the magnetic force simulates a gravitational force. We believe that future studies with MagLev systems under spaceflight and On-Earth conditions would be successful to provide evidences on gravitaxis in bacteria.

## 8. Future Industrial Applications of the Magnetic Force in Microbiological Studies

Microbial secondary metabolites that represent raw material important for medicine and/or industry amount to several thousands and include antimicrobial, antiparasitic, and antitumor compounds, growth regulators, building blocks of industrially relevant chemicals, etc. [96,97,98]. Multiple studies are devoted to industrial strain selection and/or creation of microorganisms that have been recognized as potential producers of bulk chemicals [99,100,101,102]. Besides strain selection, industrial metabolite production requires optimization of growth conditions. Real and simulated microgravity was tested to analyze how microgravity conditions affect production of important secondary metabolites. Obtained results are contradictory and demonstrated that production of secondary metabolites can be up-regulated, inhibited, or changed in its secretion depending on a substance, a strain, a growth phase, medium composition, and a microgravity model used (reviewed in [4,24]). There are only a few studies where effects of the magnetic force on secondary metabolite production were studied. Liu and co-authors used the superconducting magnet-based MagLev model to study microgravity effects on production of the anthelmintic agent avermectin by *Streptomyces avermitilis* [50]. Plating bacteria on the solid medium prevented development of indirect MagLev effects observed in liquid media but provided more space for a detailed analysis of direct gravitaxis and effects of the magnetic field and its gradient. Obtained results demonstrated increased production of avermectin under MagLev conditions and provided evidence that this increase is attributed to the strong magnetic field. These results suggest that even in the absence of direct and non-direct effects of the magnetic force, the MagLev systems might improve production of secondary metabolites via magnetic field effects. 

Using MagLev bioassambler and its space analog “Organ.Aut”, we demonstrated that the bacterial aggregation due to the magnetic force within the liquid bulk up-regulates metabolic pathways leading to production of such important molecules as succinate [19]. Succinic acid is widely used in the food industry and is an important precursor of many industrially relevant chemicals used in biomedicine and biochemistry [96,97]. Bio-based production of succinic acid is a well-established process that includes different natural and genetically-modified microorganisms. Succinic acid accumulates as a final product under anaerobic conditions, and a number of obligate anaerobes, such as *Anaerobiospirillum succiniciproducens, Actinobacillus succinogenes*, *Mannheimia succiniciproducens*, were described as industrially important producers of the succinic acid [103,104,105,106,107]. Still, easy cultivation makes genetically modified *E. coli* although it is not obligate anaerobe to be an attractive succinic acid producer [107]. Succinic acid production in *E. coli* uses dual-phase fermentations, which comprise an initial aerobic growth phase followed by an anaerobic production phase. Using MagLev system bacterial gathering in the center of the bioassmbler and corresponding changes in the oxygen availability provide a potential alternative to systems used now for dual-change fermentation of succinic acid and other potential bioproducts, which require limited oxygen availability. The unique capabilities of Maglev systems to change bacterial growth conditions on Earth and in space opens up a wide field for development of novel biotechnologies and provides ample opportunities for future research.

## 9. Conclusions

The current development in superconductive and powerful permanent magnets offers unique possibilities to create the magnetic force strong enough to provide cell movement, assembly and congregation. In microbial on-Earth studies, the strong magnetic force allows for creating MagLev systems that can be widely used in practical applications, such as test-systems for bacterial separation and identification, characterization of antibiotic resistance, and formation of non-attached bacterial aggregates. Application of magnetic force under spaceflight conditions is only at its beginning. Still, initial results suggest that the introduction of MagLev systems into real microgravity conditions opens a wide field for future research and practical applications from models of simulated gravity to bacterial factories producing substances to meet the various needs of mankind. 

## Data Availability

Not applicable.
